# Cutting-edge issues in sympathetic ophthalmia approach and six case reports: a T cell-mediated autoimmune response

**DOI:** 10.5935/0004-2749.2022-0142

**Published:** 2024-04-15

**Authors:** Fernando Henrique Flores Teixeira, Thiago Sande Miguel, Danielle Marcello Soares, Juliana Rocha, Ana Luiza Biancardi, Francisco Assis de Andrade, André Luiz Land Curi

**Affiliations:** 1 Laboratory of Infectious Diseases in Ophthalmology, Instituto Nacional Evandro Chagas, Rio de Janeiro, RJ, Brazil; 2 Ophthalmology Department, Universidade Federal Fluminense, Rio de Janeiro, RJ, Brazil; 3 Ophthalmology Department, Centro de Estudos e Pesquisas Oculistas Associados, Rio de Janeiro, RJ, Brazil; 4 Ophthalmology Department, Universidade Federal do Rio de Janeiro, Rio de Janeiro, RJ, Brazil; 5 Discipline of Uveitis and Autoimmune Diseases, Centro de Estudos e Pesquisas Oculistas Associados, Rio de Janeiro, RJ, Brazil

**Keywords:** Ophthalmia, sympathetic, Autoimmunity, Immuno-suppression therapy, Immunosuppressive agents/therapeutic use, Eye enucleation, Eye evisceration, Humans, Case reports, Oftalmia simpática, Autoimunidade, Terapia de imunossupressão, Imunossupressores/uso terapêutico, Enucleação ocular, Evisceração do olho, Humanos, Relato de casos

## Abstract

Sympathetic ophthalmia is a rare and potentially devastating bilateral diffuse granulomatous panuveitis. It is caused by surgical or non-surgical eye injuries and is an uncommon and serious complication of trauma. It is diagnosed clinically and supported by imaging examinations such as ocular ultrasonography and optical coherence tomography. Its treatment consists of immunosuppressive therapy with steroids and sometimes steroid-sparing drugs, such as cyclosporine, azathioprine, cyclophosphamide, and mycophenolate mofetil. Fast and effective management with systemic immunosuppressive agents allows for disease control and achievement of good visual acuity in the sympathizing eye. By contrast, enucleation should be considered only in situations where the injured eye has no light perception or in the presence of severe trauma. In addition to a bibliographic review of this topic, we report six cases involving different immunosuppressive and surgical treatment modalities.

## Abstract (pt)

A oftalmia simpática consiste em uma panuveíte granulomatosa bilateral rara e potencialmente devastadora, ocorrendo geralmente após trauma ocular cirúrgico ou não cirúrgico. O diagnóstico é baseado em aspectos clínicos e apoiado por exames de imagem, como ultrassonografia ocular e tomografia de coerência óptica. O tratamento consiste em terapia imunossupressora com esteróides e, eventualmente, drogas poupadoras de esteróides, como ciclosporina, azatioprina, ciclofosfamida e micofonato de mofetila. O manejo rápido e eficaz com agentes imunossupressores sistêmicos permite o controle da doença e a obtenção de boa acuidade visual no olho simpatizante. A enucleação, por outro lado, poderia ser considerada apenas em situações em que o olho lesado não tem percepção luminosa ou há trauma grave. Além de uma revisão bibliográfica sobre o tema, foi relatada uma série de 6 casos com diferentes modalidades de tratamento imunossupressor e cirúrgico.

## INTRODUCTION

Sympathetic ophthalmia (SO) consists of noninfectious endogenous uveitis characterized by bilateral panuveitis. It is an extremely rare clinical condition with uveal damage, invariably of traumatic or surgical origin^(1^,^2)^. The injured eye is known as the exciting eye and the opposite as the sympathizing eye, in which inflammation develops after days or years. The time from eye injury to SO onset varies widely from days to decades, with 80% and 90% of cases occurring within 3 months and 1 year after the injury, respectively^(3)^. Imaging tests, such as fluorescein angiography (FA), indocyanine green, B-mode ocular ultrasonography, and optical coherence tomography (OCT), can assist in the diagnosis, and laboratory tests should be performed to rule out infectious uveitis^(2)^.

Sympathetic ophthalmia can present as posterior segment inflammation, which is manifested as optic nerve edema, exudative retinal detachment, and anterior granulomatous reaction with mutton fat keratic precipitates (KPs) in severe and chronic recurrent cases^(2^,^4)^. Although uncommon, SO is a serious eye disease that can lead to blindness, both in the traumatized or arousing eye and the contralateral or sympathizing eye, constituting one of the most feared complications of any ophthalmic surgery^(1^,^2)^. Corticosteroid therapy is considered the basis of treatment after SO onset. Furthermore, immunomodulators such as cyclophosphamide, azathioprine, cyclosporine, tacrolimus, and mycophenolate mofetil should be considered when steroid therapy is unsuccessful^(1^,^2^,^3)^. The surgical approach remains controversial and should be proposed in the case of blindness or pain in the provocative eye because the visual prognosis of the sympathetic eye is variable. Furthermore, late enucleation is not beneficial^(5^,^6)^.

### Epidemiology

SO accounts for approximately 1%-2% of all uveitis cases. However, given its rarity, the exact incidence is difficult to establish, and the diagnosis is based on clinical findings and not on serological or histopathological tests^(5)^. Moreover, accurate epidemiological information is unknown because of the lack of histopathological evidence, difficulty in studying large case series, and possibility of cases not being correctly diagnosed. Based on estimates, the incidence ranges from 0.2% to 0.5% and 0.01% after penetrating eye injuries and intraocular surgeries, respectively^(3^,^4^,^5)^. The incidence of SO has markedly decreased in recent decades because of significant improvements in the prevention and control of eye injuries^(2^,^3)^. In addition to vitreoretinal surgery, other intraocular procedures such as cataract surgery, evisceration, paracentesis, and iridectomy are important risk factors. Furthermore, no racial or age predisposition was reported. The incidence is the same in men and women after surgery; however, it is more common in men after trauma because of the higher frequency of eye damage in this group^(3^,^5^,^6)^.

### Pathophysiology

The etiology of SO is not clearly understood. The most accepted theory is that cellular immunity can be directed against uveal, retinal, or surface antigens shared by photoreceptors, retinal pigment epithelium (RPE), and choroidal melanocytes^(1^,^2)^. In penetrating lesions accompanied by uveal tissue prolapse, there is a contact with the conjunctival lymphatics, which culminates in a sensitization reaction through regional lymph nodes to uveoretinal antigens. In addition, activated T-helper lymphocytes affect and perpetuate the final immune attack^(3^,^4^,^5)^. These mechanisms lead to a break in ocular immune privilege, favoring SO development.

Histiocytes and epithelioid cells penetrate the immunoreactive sites adjacent to the uvea and become part of the yellowish-white choroidal lesions in the posterior pole, equator, and middle periphery of the retina, known as Dalen-Fuchs nodules. Inflammation and damage can occur in cases where T cells bypass the RPE and enter the retina^(4^,^7)^. Genetic predisposition influences disease development, particularly the human leukocyte antigen (HLA). The expression of the HLA-A11 antigen, as well as HLA-DRB, DQA1, and DQB1, has been associated with SO. Similar associations were observed among HLA-D4, DQw3, DRw43, and Vogt-Koyanagi-Harada (VKH) syndrome^(1^,^4^,^5)^. Although an antigen can stimulate the immune response, no circulating antibodies directed against intraocular tissue have yet been found^(3)^.

### Clinical features

Regarding severity, SO involves a wide spectrum of signs and symptoms, resulting in mild to significant visual loss. SO also occurs within 3 months and 1 year after the injury in 80% and 90% of the cases, respectively^(3)^. Furthermore, patients report an insidious onset of visual blurring, pain, epiphora, and photophobia in the sympathizing eye, which is accompanied by conjunctival injection and granulomatous reaction in the anterior chamber, with mutton fat KPs in the corneal endothelium^(4^,^5^,^8)^. The anterior chamber may have a relatively mild reaction, and inflammation may be non-granulomatous. However, severe involvement can lead to posterior synechiae formation. The blockade of inflammatory cells in the trabecular meshwork and inflammation of the ciliary body may increase the intraocular pressure, which may further reduce the secondary involvement of the ciliary body^(5^,^9^,^10)^.

The extent of inflammation varies in the posterior segment. Moderate-to-severe vitreitis with yellowish-white choroidal lesions in the posterior pole, equator, and middle periphery of the retina, known as Dalen-Fuchs nodules, may be present. Other granulomatous inflammatory ophthalmic pathologies may also present with Dalen-Fuchs nodules, including VKH syndrome and sarcoidosis^(6^,^7^,^9)^. The inflammation can also lead to serous retinal detachment (SRD); optic nerve edema; cataracts; glaucoma; choroidal neovascularization; subretinal fibrosis; optic nerve, retinal, or choroidal atrophy; and phthisis bulbi^(5^,^6^,^7^,^11)^. Indirect ophthalmoscopy is useful for monitoring the disease course^(5^,^6^,^12^,^13)^. Moreover, SO is self-limited in some cases; however, in most cases, inflammation persists for many years, with periods of acute exacerbation and recurrence^(5^,^6^,^7)^.

### Diagnosis

SO cannot be diagnosed easily. A history of penetrating lesions and bilateral uveitis is an etiological basis^(6^,^7^,^11)^. In addition, no laboratory studies have established a diagnosis. However, targeted clinical tests can be used to rule out other entities with a similar clinical presentation^(4^,^8^,^9)^. In addition to laboratory tests, imaging examinations such as B-mode ocular ultrasonography, OCT, FA, and indocyanine green angiography can elucidate the condition and rule out infectious causes of uveitis^(6^,^10^,^11^,^13)^. In the acute phase, FA demonstrates multiple sites of hyperfluorescent leakage in the RPE during the venous phase that persisted in the late stages. The dye may spread from these areas. In severe cases, exudative areas tend to clump together in large portions of exudative retinal detachment^(7^,^11^,^12)^.

Occasionally, late staining of the optic nerve head is observed even in the absence of clinical papillitis or edema^(5^,^9^,^10^,^13)^. As the process predominantly involves the choroid, indocyanine green angiography can complement FA both for diagnosis and assessment of treatment response. Indocyanine green angiographies showed multifocal hypofluorescent spots that became more prominent as the test progressed^(11^,^12^,^14)^. OCT revealed disorganization and thinning of the inner retina, pronounced disintegration of the RPE and choriocapillaris, and choroidal and RPE thickening. Furthermore, ultrasonography can reveal choroidal thickening and retinal detachment^(9^,^10^,^13)^. Thus, OCT is important in detecting SO development before typical findings occur. Small choroidal lesions can initially be neglected, especially in patients with asymptomatic cases, insidious onset, or recurrence. Therefore, frequent OCT of both eyes (OU) is recommended, and careful OCT evaluation can speed up early treatment when indicated^(8^,^9^,^10^,^12)^.

### Case Reports

Herein, we report six cases of SO. Their clinical signs, symptoms, treatment modalities, and follow-up are highlighted in table 1.

**Table 1:** Clinical features of the six patients

	Eye trauma	Symptoms	Signs	Initial visual acuity	Treatment	Final visual acuity	Follow-up
**Case 1**	Complicated cataract surgery in OS	Low visual acuity	Hyperemic optic disc and serous retinal detachments in OD	OD: 20/20 OS: luminous perception	Prednisone 10 mg/day orally	OD: 20/20 OS: Luminous perception	Referred to glaucoma service
**Case 2**	Automobile accident with ocular trauma in OS	Low visual acuity	Optic disc edema and serous retinal detachment in the OD	OD: counting fingers OS: counting fingers	Prednisone 1 mg/kg/day orally	OD: 20/20 OS: 20/20	No recurrence for nearly 1 year
**Case 3**	Piercing trauma (firearm projectile) in OS 8 years ago	Low visual acuity	Vitiligo, granulomatous keratic precipitates, and vitritis in the OD	OD: 20/25 OS: no luminous perception	Prednisone 0.5 mg/ kg/day orally and Cyclosporine 3 mg/kg/day orally	OD: 20/25 OS: No luminous perception	No recurrence for 2 years
**Case 4**	Complicated cataract surgery in OS	Low visual acuity	Optic disc edema and serous retinal detachment in the OD	OD: hand motion OS: no luminous perception	Prednisone 1 mg/kg/day orally	OD: 20/25 OS: No luminous perception	No recurrence for 8 years
**Case 5**	Penetrating keratoplasty on the OS 3 weeks ago	Low visual acuity, headache and nausea	Bilateral optic disc hyperemia and multifocal serous retinal detachments	OD: 20/400 OS: counting fingers	Prednisone 1.5 mg/kg/day orally	OD: 20/20 OS: 20/200	No recurrence for 11 months
**Case 6**	Perforating trauma to the OS 30 days before	Low visual acuity	Poliosis, vitiligo, and sunset glow fundus in OU	20/160 in OU	Prednisone 5 mg/day orally and azathioprine 1.5 mg/kg/day orally	OD: 20/100 OS: 20/32	No recurrence for 12 years

OD= right eye; OS= left eye; OU= both eyes.

### Case 1

**Figure 1:**
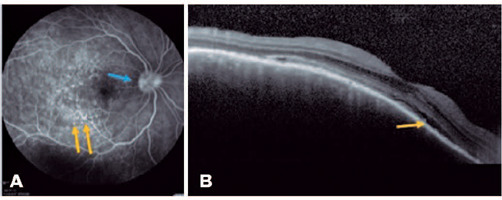
(A) Right eye: fluorescein angiography showing dye leakage in the optic disc (papillitis; blue arrow) and multiple pinpoints within the posterior pole (yellow arrows). (B). Right eye: macular OCT showing SRD (yellow arrow). Images provided by INI/Fiocruz.

A 47-year-old woman was referred to our hospital for a follow-up of SO. She underwent complicated cataract surgery with retinal detachment in the left eye (OS) preceding the condition and was treated with dexamethasone 1 mg/mL eye drops in OU, brimonidine tartrate 2 mg/mL combined with timolol maleate 5 mg/mL bid in the OS, and prednisone 10 mg/day for 4 months. Ophthalmological examination revealed visual acuity (VA) of 20/20 in the right eye (OD) and luminous perception in the OS. Biomicroscopy revealed OD without changes, and in the OS, marked flare (3+/4+), without cells and bombé iris, was observed. The intraocular pressure was 18 mmHg in the OD, and the OS presented with hypotonia. Fundoscopy revealed a hyperemic optic disc and small SRD areas at the posterior pole that also involved the subfoveal region. FA revealed multiple pinpoints dispersed throughout the posterior pole (Figure 1A), and OCT revealed SRD (Figure 1B). The patient was followed up for 3 years, with gradual tapering of prednisone dosage until its suspension in 2015. OD fundoscopy demonstrated a “sunset glow fundus” appearance and Dalen–Fuchs nodules in the periphery; however, OS examination was impossible. The patient subsequently developed secondary glaucoma in the OD and was referred to a specialized service.

### Case 2

**Figure 2:**
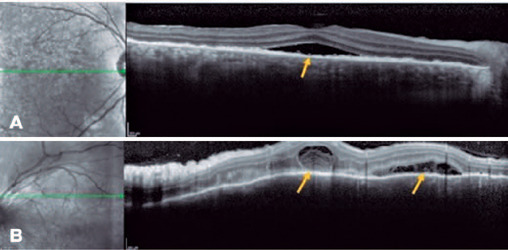
(A). Right eye: macular OCT showing a subfoveal SRD (yellow arrow). (B) Left eye: OCT showing SRDs with fibrinoid material (yellow arrow). Images provided by INI/Fiocruz.

A 43-year-old man presented with low insidious and painless VA in OU. Ophthalmological examination revealed optic disc edema in the OD, and OCT showed SRD involving the subfoveal region (Figure 2A) along with the superior regions of the posterior pole, the latter with septa and fibrinoid material (Figure 2B). On OCT, the OS demonstrated irregularity in the RPE. The patient denied a previous trauma history; however, biomicroscopy revealed a small inferior leukoma with an anterior synechia attached to it. Infectious diseases were ruled out, and in the anamnesis review, the patient recalled having experienced an automobile accident but was unaware of the ocular trauma. Systemic treatment with the oral administration of prednisone (1 mg/kg/day) was initiated in a slowly tapering dosage. The patient had improved VA and fundoscopic findings and was followed up for nearly 1 year without recurrence.

### Case 3

**Figure 3:**
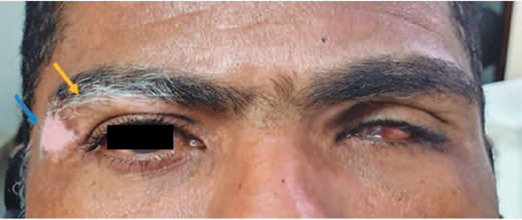
Right eye: Upper and lateral eyelid vitiligo (blue arrow) and poliosis (yellow arrow). Left eye: Poliosis and *phthisis bulbi*. Image provided by INI/Fiocruz.

A 40-year-old man was referred to the National Institute of Infectious Diseases, Fiocruz, complaining of low progressive and painless VA for 2 months. He had been using dexamethasone 1 mg/mL eye drops tid and prednisone 0.5 mg/kg/day. Eight years ago, he was pierced by a firearm projectile, resulting in an amaurotic OS. On ectoscopy, he presented with vitiligo in the upper right eyelid (Figure 3). Ophthalmological examination revealed a VA of 20/25 in the OD, biomicroscopy revealed sparse granulomatous KPs in the OD, and absence of cells/flare, posterior synechiae, incipient corticonuclear cataract, and phthisis bulbi in the OS. Fundoscopy of the OD was impossible because of miosis and subsequent synechiae. Ultrasonography of the OD revealed only fine vitreous opacities. Owing to a history of perforating trauma and vitiligo-associated granulomatous uveitis, SO was considered. The drug dosage was gradually reduced until the suspension was performed, and no complications were observed during the 3-year follow-up. After this period, the patient returned reporting floaters. Ophthalmological examination revealed VA maintained in the OD, and biomicroscopy revealed 1+ cells without flares or KPs. Considering the severity of the underlying disease in a single eye, oral readministration of prednisone (0.5 mg/kg/day), topical application of dexamethasone (1 mg/mL), and tropicamide (1 mg/mL) were initiated. To proceed with the gradual reduction of prednisone, azathioprine (1 mg/kg/day) was introduced. However, this led to drug hepatitis in a short period, resulting in its suspension. Azathioprine was replaced by cyclosporine 3 mg/kg/day with the discontinuation of oral prednisone treatment, after which the disease was controlled. The patient has been followed up for 2 years without recurrences.

### Case 4

A 61-year-old woman, born in Rio de Janeiro, presented with VA of hand motion in the OD for a week. She had a history of cataract surgery, vitreous loss, and iris exposure. She had no luminous perception in the OS. Fundoscopy revealed optic disc edema and SRD in the OD, which was impossible in OS. Infectious diseases were ruled out, and SO was diagnosed. Prednisone (1 mg/kg/day) was administered orally for 3 days, which led to a significant improvement in VA, and oral prednisone dosage was gradually tapered. Oral medication was maintained for approximately 2 years at a dose of 5 mg/day until the treatment was suspended. At that time, the VA was 20/25 in the OD, and no luminous perception caused by phthisis bulbi in the OS was observed. Fundoscopy revealed a “sunset glow fundus” at the OD. The patient had been off systemic medications and had no recurrence signs for 8 years.

### Case 5

**Figure 4:**
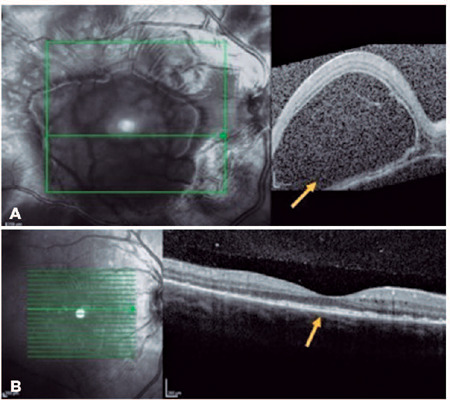
(A) Right eye: macular OCT showing extensive bacillary retinal detachment (yellow arrow). (B) Right eye: macular OCT 4 months after onset revealing improvement of SRD (yellow arrow). Images provided by MD Juliana Rocha.

**Figure 5:**
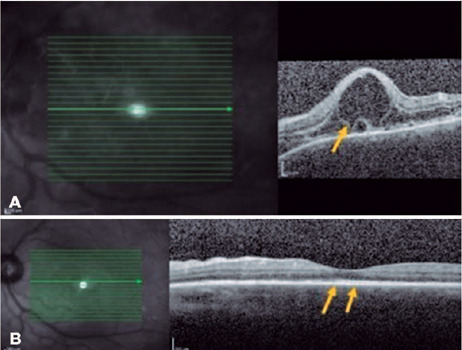
(A) Left eye: macular OCT showing bacillary retinal detachment (yellow arrow). (B) Left eye: macular OCT 4 months after onset (yellow arrows). Images provided by MD Juliana Rocha.

A 23-year-old woman presented with a sudden onset of reduced vision in her OD since the past 24 h, which was associated with headache and nausea. She had a history of bacterial keratitis in the OS caused by contact lens misuse that led to corneal thinning with three episodes of corneal perforation; the first two were successfully treated by cyanoacrylate glue, and the last one was complicated with persistent iris herniation. Subsequently, she underwent penetrating keratoplasty of the OS, 3 weeks before the OD event. Ophthalmic examination revealed a VA of 20/400 in the OD and counting fingers in the OS. Anterior-segment evaluation showed 3+ cells in the OD anterior chamber. Corneal edema restricted the evaluation of the anterior chamber and vitreous of the OS. Fundus examination revealed bilateral optic disc hyperemia and multifocal SRD. Ancillary tests were performed. FA showed bilateral diffuse early pinpoints and later pooling within the SRD. In addition, a remarkable optic disc hyperfluorescence was observed in the OD. Spectral-domain OCT revealed multifocal serous and bacillary retinal detachments and pigmented epithelial detachment (Figures 4A and 5A). A diagnosis of SO was made, and the patient was treated with high-dose systemic steroids (oral administration of prednisone 1.5 mg/kg/day), intensive topical steroids, and topical atropine (1%). After 14 days of treatment, subretinal fluid improved in OU (Figures 4B and 5B). Oral prednisone dosage was gradually tapered, and 4 months after the onset, at a dosage of 0.45 mg/kg/day, the best-corrected VA values were 20/20 and 20/200 in the OD and OS, respectively. Eleven months after the onset of inflammation, the patient’s condition was stable, without any oral or topical treatment. The sunset glow retinal changes remained without any evidence of inflammation recurrence.

### Case 6

**Figure 6:**
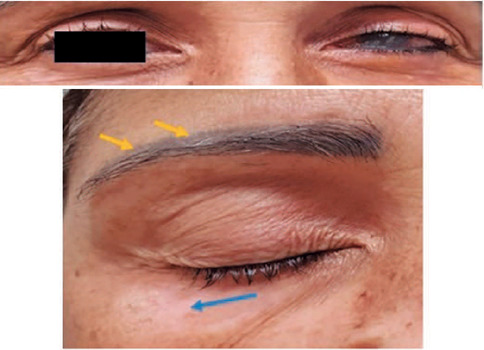
Left eye: ectoscopy showing poliosis (yellow arrow) and vitiligo (blue arrow). Image provided by INI/Fiocruz.

**Figure 7:**
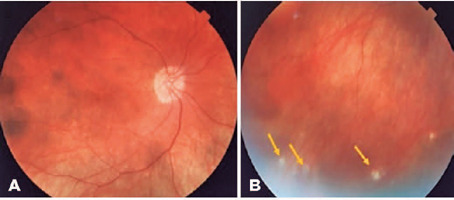
(A) Right eye: retinography showing a sunset glow fundus. (B) Right eye: retinography of the inferior retina showing Dalen–Fuchs nodules (yellow arrows). Images provided by INI/Fiocruz.

A 46-year-old woman sought care for a history of perforating trauma to the OS in 2006 and had subsequent low VA in the OD 30 days later. After excluding infectious causes, SO was diagnosed. She was referred to the ophthalmology service of the National Institute of Infectious Diseases Evandro Chagas-Oswaldo Cruz Foundation in 2009, where she received timolol maleate 5 mg/mL eye drops bid, brimonidine tartrate 2 mg/mL eye drops bid, travoprost 0.04 mg/mL eye drops qd, azathioprine 1.5 mg/kg/day orally, and prednisone 5 mg/day orally. On ectoscopy, the patient had poliosis and vitiligo (Figure 6). Ophthalmological examination revealed a VA of 20/160 in OU, biomicroscopy did not detect alterations in the OD, and corneal scar corresponding to trauma, posterior subcapsular cataract 1+/4+, posterior synechia, and absence of inflammatory reaction in the OS. Fundoscopy revealed physiological cupping of the optic nerve and diffuse atrophy of the RPE with pigment mobilization, characterizing the “sunset glow fundus” in OU (Figures 7A and 7B). Regarding treatment, 5 mg prednisone was prescribed on alternate days, with the maintenance of other medications. The patient has been followed up for 12 years, and in 2021, she presented with a VA of 20/100 and 20/32 in OD and OS, respectively, without immunosuppressants and evolutionary changes, and was on travoprost (0.04 mg/mL) eye drops qd for OU.

### Differential diagnosis

Differential diagnosis must include all diseases that present with panuveitis, paying attention to the history of penetrating lesions such as trauma and surgery^(14^,^15^,^16^,^17)^. Lymphoma, syphilis, tuberculosis, and sarcoidosis must be ruled out because they demonstrate several small foci of choroiditis and vitreous cellularity. These conditions are usually accompanied by constitutional signs and symptoms of the underlying systemic disease. Therefore, the analysis of the clinical picture associated with appropriate diagnostic tests for each pathology distinguishes these comorbidities from SO^(14^,^17^,^18)^.

VKH syndrome, a systemic disease, involves tissues that contain melanin. It is characterized by bilateral, chronic, and granulomatous panuveitis associated with variable manifestations of neurological, auditory, and cutaneous impairment^(17^,^18)^. Bilateral eye involvement is necessary to characterize the diagnosis, although it may be asymmetric, and there must be no previous history of eye surgery or penetrating ocular trauma. Iridocyclitis, vitreitis, optic disc edema, hyperemia, choroidal thickening, and neurosensory retinal detachment are the most common symptoms^(14^,^15^,^17)^.

Differentiating SO from VKH syndrome is difficult. However, in the VKH syndrome, no patients had a history of surgery or trauma, and the condition presents as bilateral granulomatous panuveitis with prominent choroidal involvement. In addition, VKH is more prevalent in certain racial and ethnic groups, showing more frequent cutaneous changes such as vitiligo, alopecia, and poliosis, and neurological symptoms such as tinnitus, headache, or altered consciousness, in addition to pleocytosis in the cerebrospinal fluid^(14^,^19^,^20)^. Both are autoimmune pathologies that target melanin-bearing cells and are characterized by immune dysregulation; thus, the two disorders have distinct etiologies but with similar ocular and systemic manifestations^(12^,^14^,^16)^.

### Treatment strategies

Sympathetic ophthalmia is a potentially threatening disease with high rates of visual loss and requires immediate evaluation and treatment. With the advances in and availability of immunotherapy, the visual prognosis of SO is relatively good^(5^,^6^,^13)^. Immunosuppressive therapy is usually initiated when the patient demonstrates inadequate response to systemic steroids or when the dose required for systemic steroids is reduced. A gradual escalation of multiple immunosuppressive agents is employed when the first-line treatment is insufficient^(19^,^20^,^21)^. Topical and systemic corticosteroids are first-line therapies for SO. The most widely used initial treatment is oral administration of prednisone, which is given in high doses of 1.0-2.0 mg/kg/day and gradually reduced in 3-4 months. In severe cases, pulse therapy can be used with an intravenous steroid (methylprednisolone 1.0 g/day) for 3 or 5 days. Synthetic disease-modifying antirheumatic drugs, such as azathioprine, cyclosporine, tacrolimus, and mycophenolate, should be considered steroid-sparing agents or when a satisfactory inflammatory response is not obtained with corticosteroids alone. If these approaches fail, biological therapy (such as adalimumab and infliximab) is pursued. In severe inflammation, techniques such as intravenous administration of cyclophosphamide may be used, and if the patient is in remission for 6-12 months, the treatment is subsequently switched to a less toxic one ^(7)^.

Topical cycloplegic agents and corticosteroids are administered to prevent anterior uveitis complications, such as anterior synechiae and anterior-segment inflammatory reaction^(1^,^8^,^22^,^23^,^24^,^25)^.

To date, no controlled clinical trials have analyzed the use of steroid-sparing immunosuppressive therapy for SO, mainly because of disease rarity. However, few reports include cases of successful control of inflammation in patients with SO who were treated with various combinations of immunosuppressive drugs^(10^,^22^,^26)^.

A surgical approach is necessary in the absence of a satisfactory therapeutic response, cases of blindness, and pain in the inciting eye^(9^,^19^,^23^,^24)^. However, this approach remains controversial^(14^,^16^,^20^,^21^,^22)^. In 2018, a multicenter retrospective cohort study revealed that enucleation of the inciting eye was not related to better visual results^(1)^, showing that enucleation decisions must be carefully considered. Not infrequently, the inciting eye can end up becoming the eye with the best vision. Certainly, enucleation should not be the first-line of treatment; however, it can be an option after other alternatives have been exhausted, especially in the case of a painful eye^(1^,^11)^.

Evisceration surgery consists of the complete removal of the intraocular contents through a corneal or scleral incision, while preserving the optic nerve^(3^,^14^,^15^,^20)^.

Although evisceration is better accepted by patients because of the greater preservation of ocular tissues, this aggressive procedure can cause trauma from both anatomical and psychological perspectives. The indications for this surgery must be careful, precise, and well-oriented^(4^,^22^,^25)^.

SO, a rare and potentially devastating bilateral panuveitis, usually occurs after surgery or penetrating trauma to one eye. A high index of suspicion is essential to guarantee an early diagnosis and the initiation of the most adequate treatment, allowing for the control of the inflammatory reaction and achieving the best possible VA. Several clinical presentations are possible, and bilateral uveitis that presented after eye surgery should alert the surgeons. Despite the lack of consensus on the ideal treat ment, most experts agree that SO requires immediate attention and treatment, both with systemic corticosteroids and immunosuppressive therapy. In conclusion, early aggressive treatment results in better outcomes. However, one-third of the cases have a vision worse than 20/200, and relapses can occur after several years; thus, long-term follow-up is essential, even in patients who presented remission after several years, because of the possibility of permanent and late sequelae.
